# Functions of Circular RNAs in Regulating Adipogenesis of Mesenchymal Stem Cells

**DOI:** 10.1155/2020/3763069

**Published:** 2020-08-01

**Authors:** Fanglin Wang, Xiang Li, Zhiyuan Li, Shoushuai Wang, Jun Fan

**Affiliations:** ^1^Department of Tissue Engineering, School of Fundamental Science, China Medical University, Shenyang, Liaoning 110122, China; ^2^Department of Cell Biology, Key Laboratory of Cell Biology, Ministry of Public Health, And Key Laboratory of Medical Cell Biology, Ministry of Education, China Medical University, Shenyang, Liaoning 110122, China

## Abstract

The mesenchymal stem cells (MSCs) are known as highly plastic stem cells and can differentiate into specialized tissues such as adipose tissue, osseous tissue, muscle tissue, and nervous tissue. The differentiation of mesenchymal stem cells is very important in regenerative medicine. Their differentiation process is regulated by signaling pathways of epigenetic, transcriptional, and posttranscriptional levels. Circular RNA (circRNA), a class of noncoding RNAs generated from protein-coding genes, plays a pivotal regulatory role in many biological processes. Accumulated studies have demonstrated that several circRNAs participate in the cell differentiation process of mesenchymal stem cells in vitro and in vivo. In the current review, characteristics and functions of circRNAs in stem cell differentiation will be discussed. The mechanism and key role of circRNAs in regulating mesenchymal stem cell differentiation, especially adipogenesis, will be reviewed and discussed. Understanding the roles of these circRNAs will present us with a more comprehensive signal path network of modulating stem cell differentiation and help us discover potential biomarkers and therapeutic targets in clinic.

## 1. Introduction

Circular RNAs are a new and intriguing class of noncoding RNA, produced from precursor mRNA (pre-mRNA), single-stranded, and covalently closed. circRNAs have been discovered for more than 40 years [1], once were described as the by-products of aberrant splicing with functional proteins [2–4]. This cognition has been changed in recent years, with the development of high-throughput sequencing technology and novel bioinformatics algorithms [5]. Thus, backspliced junctions of circRNAs can be reliably identified, through short-read paired-end RNA sequencing (RNA-seq) technology [6, 7]. By sequencing nonpolyadenylated transcriptomes, researchers find that circRNAs are generally expressed in eukaryotes. Their expression has characteristics of cell type-specific and tissue-specific [5, 8]. Although the functions of thousands of described circRNAs remain unknown, accumulated studies have already shown that circRNAs can participate in cellular activities, embryonic development, neural development, and the development of a variety of human diseases [9, 10]. With the regulatory role of circRNAs, the researchers try to find out the potential functions of circRNAs in cell differentiation of mesenchymal stem cells.

The mesenchymal stem cells can be isolated from adult tissues, including bone marrow, adipose tissue, and umbilical cord, and other sources [11]. MSCs are multipotent stromal cells with low immunogenic potential that can differentiate into a variety of unique mesenchymal cell types, such as osteoblasts, chondrocytes, and adipocytes. With the development of regenerative medicine, MSCs have been widely applied in effective cell-based therapy for tissue regeneration and repair [12–14].

This review is aimed at presenting and discussing the mechanism on how circRNAs affect adipogenic differentiation of MSCs based on existing literature. Moreover, we propose to summarize the signal path network and figure out the direction to be studied.

## 2. Adipogenic Differentiation of MSCs

Mesenchymal stem cells are a heterogeneous population of multipotent elements resident in tissues such as bone marrow, muscle, and adipose tissue, which are primarily involved in developmental and regeneration processes [15]. MSCs participate in the repair/remodeling of many tissues. They have an ability termed “plasticity” in which they differentiate into specific cell-matured phenotypes under defined conditions [16]. The control of stem cell fate has been primarily attributed to the regulation of genetic and molecular mediators which are critical determinants for the lineage decision of stem cells. Though complex signaling pathways that drive commitment and differentiation, MSCs can differentiate into the osteogenic, chondrogenic, adipogenic, or myogenic lineage.

Adipogenesis is one of the significant differentiation directions of mesenchymal stem cells, which can be regulated by transcription factors [17]. In this process, MSCs restrict their fate to the adipogenic lineage, accumulate nutrients, and become triglyceride-filled mature adipocytes. This process can be divided into two steps. In the first step, ADSCs restrict themselves to the adipocyte lineage without any morphological changes, forming a preadipocyte (commitment step). Following this commitment is the terminal differentiation step, during which specified preadipocytes undergo growth arrest, accumulating lipids and forming functional, insulin-responsive mature adipocytes [18].

### 2.1. Commitment Step

At the launch stage of adipogenesis, the expression of c-Jun and c-Fos in ADSCs is increased [19]. c-Fos binds to the AP-2 promoter and modulates AP-2 expression [20]. At this moment, binding of AP-2*α* to CCAAT/enhancer-binding protein (C/EBP) *α* represses its activity to avoid C/EBP*α* preventing preadipocytes from entering mitotic clonal expansion. The signal transducers and activators of transcription (STAT) family, especially STAT 5A and 5B, are highly expressed in this stage [21]. The induction of STAT 5A/5B in preadipocytes is accompanied by the ectopic expression of C/EBP*β* and *δ* and is also coordinately regulated with the peroxisome proliferator-activated receptors (PPAR) *γ* [22]. After 2-6 hours of induction, the expression of c-Jun, c-Fos, etc. disappeared. In this early lineage commitment process, Wnt/*β*-catenin functions as a critical initiator, suppresses the induction of adipogenesis, and regulates the cell cycle [23, 24]. Reports have shown that Wnt10b appears to have an activating role in commitment and maintains preadipocytes in an undifferentiated state through inhibition of C/EBP-*α* and PPAR*γ* [25, 26].

Following a delay of about 16–20 hours after induction, preadipocytes synchronously reenter DNA synthesis prophase of the cell cycle [27] and undergo several rounds of mitotic clonal expansion. Along with preadipocytes entering into S phase and mitotic clonal expanding, C/EBP*β* gains its DNA-binding activity. C/EBP*β* is phosphorylated twice sequentially, leading to the acquisition of DNA-binding function [28]. After 18–24 hours, the C/EBP*α* and PPAR*γ* genes are transcriptionally activated by C/EBP*β* through C/EBP regulatory elements in their proximal promoters [29]. PPAR*γ*2 and C/EBP*α* coordinately transactivate a large group of genes that produce the adipocyte phenotype. They constituted the most important signal path of adipogenic differentiation, C/EBP*β*+*δ*-PPAR*γ*-C/EBP*α* [30].

### 2.2. Terminal Differentiation Step

Preadipocytes have entered the growth inhibition phase; then, the cells immediately return to the cell cycle and enter the asexual amplification stage. While the transcriptional activity of C/EBP*β*/*δ* is enhanced by STAT 5A/5B, C/EBP*α* is increasingly expressed and achieved the highest concentration in the time of induction for 4 d. [22, 31]. The cells then exit the cell cycle losing their fibroblastic morphology and start accumulating triglyceride in the cytoplasm with the appearance and metabolic features of adipocytes [32]. Lipid accumulation drives the expression of the adipocyte fatty acid-binding protein, AP2, and mediates PPAR*γ* expression. The insulin-sensitive transporter GLUT4 also expressed increasingly, promoting the triglyceride accumulation [18]. In the terminal differentiation, the canonical Wnt signaling pathway can also regulate adipogenesis by attenuating the expression of Wnt10b [25]. Wnt family members can also activate noncanonical pathway antagonizing the canonical pathway [32]. Through Wnt5b, the noncanonical pathway inhibits adipogenesis by decreasing the transcriptional activity of PPAR*γ* and regulates insulin sensitivity of the differentiating adipocytes [33, 34]. Then activated phosphoinositide 3-kinase (PI3K)/serine/threonine protein kinases B (Akt) signaling in mesenchymal cells drives and maintains the adipogenesis around 7-28 d after induction [35]. With such complex factors regulated, adipogenic precursor cells complete triglyceride accumulation after a few days of induction, showing typical adipocyte morphology.

## 3. Characteristics and Functions of circRNA

circRNAs are generated from pre-mRNAs; most of them are arising from exons. By using the standard splice signals, two-step mechanism, and spliceosome machinery, the 3′ tail of one exon is joined to the 5′ head of an upstream exon; pre-mRNAs compose into a covalently closed and circular configuration [6, 36, 37]; besides, some circRNAs can also arise from introns [38]. The formation of circRNAs depends on the RNA-editing enzyme ADAR1 and can be facilitated by *cis*-regulatory elements and *trans*-acting factors [36, 39–42]. According to types of circularization, circRNAs can be classified into exonic circRNAs and intronic circRNAs. They are distinct and independent varieties in a generation. In recent years, researchers focus on clarifying the functions of circRNAs, which are involved in the regulation of multiple biological processes.

### 3.1. miRNA Sponge

circRNAs are usually considered one class of noncoding RNA, which mainly function as efficient microRNA (miRNA) sponge, which is involved with miRNA inhibition with regulatory potential [9, 43, 44]. Indeed, this phenomenon has been widely reported, circRNA overexpression could alleviate apoptosis and promote anabolism through the miRNA pathway [45, 46]. This route is generally considered endogenous RNA (ceRNA) and constitutes a regulatory network across the transcriptome [47, 48]. In the regulation of MSC differentiation, sponging function of circRNA has been extensively investigated. However, only a few of circRNAs were showed to serve as miRNA sponge [49, 50]. They may play a part in other functions. In addition, some of the circRNAs do not display binding sites for miRNA; future efforts should be done to elucidate the mechanisms on improving circRNAs to expose their binding sites to the corresponding miRNAs.

### 3.2. Sponging Protein

There is still a large class of circRNAs with many protein binding sites, which are essential for strong and direct interaction between protein and circRNAs. Target protein can impact circularization rates of circRNA, and circRNA could then sponge out the excess protein by binding to it [51, 52]. circRNAs function as protein sponge and are less found in regulating MSC differentiation. This function is more directly related to protein-protein interaction, which means they are significant for MSC differentiation [53].

### 3.3. Protein-Coding Function

circRNAs are generally considered “noncoding” RNAs; in fact, they also serve as templates for protein translation. A few publications provide initial evidence that circRNAs contain an open reading frame and can encode polypeptides and even a protein isoform in a splicing-dependent/cap-independent manner [10, 49, 54]. A protein-coding function of circRNAs is recently discovered; it not only corrects the wrong perception but also provides a new research area.

### 3.4. Other Functions

circRNAs can be linked to exon skipping and affect the splicing of their linear mRNA counterparts [55]. Nuclear retained circular RNAs regulate transcription of their parental genes and splicing of their linear cognates [56]. A mount of circRNA-derived pseudogenes has been identified by retrieving noncolinear backsplicing junction sequences, which demonstrates that circRNAs are resources for the derivation of pseudogenes [57]. circRNA can also act as a protein subunit associated with the holoenzyme in metabolic adaptation, assembles and stabilizes the holoenzyme complex, and maintains basal activity [58].

Based on the above studies, it enlightens us that it is necessary for exploring how circRNAs regulate MSC differentiation. Additionally, epigenetic modifications can also occur in RNA, called the epitranscriptome, which refers to stable and heritable changes in gene expression that do not alter the RNA sequence. N6-Methyladenosine (m6A) is always found to occur in the consensus sequence identified as RRACH (R = G or A; H = A, C, or U) and promote the translation of the circRNAs [59]. It is the most abundant epigenetic modification in eukaryotes and plays a significant role in autophagy and adipogenesis regulation [60]. If we combine with epigenetic modification, researches about regulating functions of circRNA in MSCs may come to a new stage.

## 4. circRNA and Regulation of MSC Differentiation

circRNAs are the key regulators of gene expression and protein functions in epigenetic regulation. As previously mentioned, the differentiation of MSC is a highly controlled process, which is regulated by both proteins and noncoding RNAs. Due to their functions in regulating epigenetic and molecular biological processes, recent research suggests that circRNAs play a considerable role in cell fate decisions of MSC differentiation [10]. Current studies demonstrate that the majority of circRNAs participate in the process of adipogenesis, myogenesis, and osteogenesis of MSCs [61, 62].

### 4.1. Adipogenesis and Osteogenesis of MSCs

Osteogenesis is thought to be most closely related to adipogenesis in the differentiation of MSCs. There is a balance between adipogenic and osteogenic differentiation processes. circRNAs regulate adipogenesis and osteogenesis by, respectively, affecting a variety of signaling pathways, but in the end, they will converge at several major transcription factors that are shared in differentiation including PPAR*γ* and WNT [63, 64].

Cerebellar degeneration-related protein 1 transcript (CDR1as), also known as circular RNA sponge for miR-7 (ciRS-7), was reported to be involved in the osteogenesis [61, 65]. CDR1as can inhibit osteogenesis and promotes adipogenesis by inhibition of miR-7 via targeting GDF5 through the MAPK signaling pathway in bone mesenchymal stem cells (BMSCs) [66]. Beyond that, many circRNAs can regulate the proliferation and osteogenesis of MSCs through sponging some specific miRNAs which are similar to their effect on adipogenesis and other differentiation [67–69].

### 4.2. Myogenesis and Neuronal Differentiation

circRNAs are also abundant in skeletal muscle tissue, and their expression levels can regulate muscle development, ageing, and differentiation. As described above, CDR1as is involved in various directions of differentiation in MSC. In skeletal muscle satellite cells (SMSCs), it subsequently activates myogenesis through sponging miR-7 [70, 71]. Several circRNAs also work in the same way by sponging miRNAs to promote or repress the myogenic differentiation of MSCs, such as circHIPK3 and circ-FoxO3 [72–74]. Other than that, circRNAs have been identified to be differentially expressed in different differentiation stages of neural stem cells (NSCs). It is likely that some specific circRNAs resulted in the corresponding expression of mRNA and involved in neuronal differentiation [75].

These shreds of evidence suggest that circRNAs play a significant role in regulating MSC differentiation. The current review is aimed at highlighting the regulation of adipogenic differentiation of MSCs by circRNAs.

## 5. circRNAs Regulate Adipogenesis

The pathways of circRNAs regulating osteogenesis and myogenesis are closely related to adipogenic differentiation. Even certain circRNAs play a multiplicative role that antagonizes among these directions of differentiation. Therefore, circRNA regulating cell differentiation of MSC is an exchange network that each differentiation direction is closely related. It suggests that the perspective of adipogenesis will contribute to understanding the landscape of the cell differentiation network of mesenchymal stem cells. The research methods of circRNAs regulating osteogenesis and myogenesis can also be used in the study on circRNA regulating network of adipogenesis. It is efficient for gene chip technology and bioinformation analysis to be used to preliminary screen. Combined with molecular biology experiment, the pathway of circRNA regulating adipogenesis could be basically verified. According to the regulatory network of circRNAs in adipogenesis, researchers can explore more directions of MSCs in clinical transformation.

Recent studies have demonstrated a few circRNAs acting as a miRNA sponge to regulate adipogenic differentiation, while other circRNAs can also compete with proteins that participate in the regulation of adipogenesis. Therefore, we emphasized the regulatory network of circRNAs in regulating adipogenesis (Table 1).

### 5.1. CDR1as–miR-7-5p–WNT5B

Confronting research discovers that circRNA, hsa_circ_0001946 (CDR1as), may play a crucial role in adipogenic/osteogenic differentiation process via CDR1as–miR-7-5p–WNT5B axis in BMSCs [61]. CDR1as has been reported to affect the expression of target genes and plays an important role in the pathogenesis via adsorbing miR-7-5p [84–86]. miR-7-5p can be sponged by CDR1as [66] and target WNT5B-3′UTR [61]. Experiments show that the upregulation of CDR1as will promote the expression of WNT5B via competitively harboring miR-7-5p. WNT5B, a member of the WNT family, can inhibit *β*-catenin in the WNT/*β*-catenin signaling pathway [87, 88]. Downregulation of *β*-catenin can promote the expression of PPAR*γ*, which promotes adipogenic differentiation and inhibits osteogenic differentiation in BMSCs [61]. In 3T3-L1 preadipocytes, low expression of *β*-catenin can also promote adipogenic differentiation and inhibit osteoclast differentiation by inducing the expression of PPAR*γ* [89, 90]. However, further studies will be needed to demonstrate the effects of circRNAs on BMSC adipogenic differentiation in vivo.

### 5.2. CircH19–PTBP1

Hsa_circ_0095570 derived from H19 pre-RNA, also called circH19, has putative binding sites with RNA-binding protein polypyrimidine tract-binding protein 1 (PTBP1) [53]. PTBP1, also known as hnRNP I, belongs to a subfamily of heterogeneous nuclear ribonucleoproteins (hnRNPs), which moves rapidly between the nucleus and cytoplasm as a shuttling protein [91]. On the asexual amplification stage, a basic helix-loop-helix transcription factor is expressed in adipocytes during adipogenesis and determination, called sterol-regulatory element-binding protein 1 (SREBP1, ADD1). One of its isoforms, SREBP-1c, contributes to the generation of PPAR*γ* ligands and promotes energy mobilization with phosphorylation by MAPK [92, 93]. A previous study demonstrated that PTBP1 played an important role in the cleavage of SREBP1 precursor and translocation of nSREBP1 protein [94]. circH19 might interact with PTBP1 to block the function of PTBP1, resulting in the inhibition of SREBP1 precursor cleavage. To sum up, has_circH19 suppresses PTBP1 and decreases the cleavage of the SREBP1 precursor, thus inhibiting the translocation of nSREBP1 to the nucleus. The inhibition of circH19 can promote the transcription of lipid-related genes, leading to lipid accumulation in hADSCs [53]. In other words, circH19 can function as an inhibitor of PTBP1 and prevents hADSCs from transforming into adipocytes with enhanced ability to absorb lipids. This is the first time to demonstrate that circRNA regulates MSC adipogenesis by sponging protein. High levels of hsa_circH19 is an independent risk factor for metabolic syndrome. So, the expression of hsa_circH19 might be related with lipid metabolism in adipose tissue from patients of metabolic syndrome.

### 5.3. CircFOXP1–miR-17-3p/miR127-5p

CircFOXP1 originates from the forkhead box (FOX) P1 gene, which is related to the maintenance of BMSC identity and regulation of differentiation. CircFOXP1 acts as an essential gatekeeper of BMSC identity, which can promote proliferation and differentiation by target sponging miR-17-3p and miR-127-5p [76]. The combined action of miR-17-3p and miR-127-5p may regulate growth, survival, and balance between undifferentiated and differentiated MSCs through epidermal growth factor receptor (EGFR) and noncanonical Wnt signaling [76, 95–97]. In BMSCs, elevated levels of circFOXP1 can preserve the BMSC multipotent state by sponging multiple miRNAs, sustain noncanonical via Wnt5b, and consequently inhibit the canonical Wnt pathway via Wnt3a [76]. This functional interaction is fundamental to inhibit miRNA activity and avoid interference of signaling cascades associated with stemness and differentiation. CircFOXP1 should be regarded as a regulator of sustaining mesenchymal stem cell identity and the capacity of MSCs to differentiate into the adipocytic lineage [76]. Knockdown circFOXP1 in MSCs will inhibit adipogenic differentiation and decrease accumulation of intracellular lipid droplets.

### 5.4. circRNA 2: 27713879|27755789 and circRNA 2: 240822115|240867796

Estrogen receptor (ER) *β* is structurally and functionally related to isoforms of ER. Its expression is increased during osteoblast differentiation [98]. It was shown that ER*β* was capable of upregulating the expression levels of osteogenesis-related markers and inducing the osteogenic differentiation of MC3T3-E1 [77]. Recently, the experiment demonstrates that ER*β* may regulate the expression levels of miR-328, miR-23a-5p, and miR-326 via circRNA 2: 27713879|27755789 and circRNA 2: 240822115|240867796 and consequently impact on the balance between osteogenic differentiation and adipogenic differentiation. In their experiment, circRNA 2: 27713879|27755789 and circRNA 2: 240822115|240867796 were identified to target miR-328 [78], while miR-328 can upregulate the expression of C/EBP*α* to inhibit cell proliferation and is involved in the dynamic balance between osteogenesis and adipogenesis [78]. circRNA 2: 27713879|27755789 and circRNA 2: 240822115|240867796 can also target miR-23a-5p and miR-326 as miRNA sponge by regulating the TGF-*β* signaling pathway and serve as an inhibitor of adipogenic differentiation [78, 99, 100]. The above study shows that circRNAs can work together and affect several kinds of miRNA to achieve its regulating potential. This discovery is very helpful for contributing the circRNA regulating network in MSC differentiation.

### 5.5. circRNA-11897–miR-27a/miR-27b–PPAR*γ*

miR-27 is an antiadipogenic microRNA partly by targeting prohibitin (PHB) and impairing mitochondrial function. Ectopic expression of miR-27a or miR-27b impaired mitochondrial biogenesis, structural integrity, and complex I activity accompanied by excessive reactive oxygen species production [101]. miR-27a can accelerate the hydrolysis of triglyceride and suppress adipocyte differentiation by repressing the expression of PPAR*γ* [102]. Via identification and characterization of circRNAs, researchers find that circRNA-11897 can bind miR-27a and miR-27b [79]. By sponging miR-27a and miR-27b, circRNA-11897 regulates adipogenic differentiation and lipid metabolism of adipocyte in the subcutaneous adipose tissue [79]. Besides, the target genes of circRNA-11897 are also enriched in biological processes, which are related to lipid metabolisms such as fatty acid biosynthetic process, MAPK cascade reaction, extracellular-regulated protein kinase (ERK)1 and ERK2 cascade reaction, and cell proliferation [79]. In the early stage of adipogenic differentiation, the activated ERK signaling pathway can induce the expression of C/EBP and PPAR*γ* and induce adipogenic differentiation of 3T3-L1 preadipocytes [103]. In a later stage, ERK1/2 is phosphorylated, and PPAR*γ* is inactivated. Thus, preadipocyte differentiation is inhibited [104].

### 5.6. circRNA-26852–miR874-PPAR*α* and circRNA-26852–miR486–FOXO1

miR-874 and miR-486 are the target genes of circRNA-26852, which are enriched in biological processes of adipocyte in the subcutaneous adipose tissue. These miRNAs are related to fat deposition and lipid metabolism, such as regulation of triglyceride catabolic process, negative regulation of lipid storage, and phosphatidic acid biosynthetic process [79]. miR-874 can regulate lipid metabolism, glycerophospholipid metabolism, adipocyte differentiation, and glucose metabolism by inhibiting the expression of PPAR pathway-related genes, PPAR*α* [105, 106]. miR-486 can inhibit the transcription factor FOXO1 and plays a role in insulin functioning and triglyceride metabolism. Therefore, circRNA-26852 working as competing for endogenous RNAs of miR-874 and miR-486 may participate in adipogenic differentiation and lipid metabolism. The pathway enrichment analysis shows that the target genes of circRNA-26852 are enriched in the PPAR signaling pathway and transforming growth factor-*β* (TGF*β*) signaling pathway. Through these two signaling pathways, circRNA-26852 regulates the transformation of mesenchymal stem cells into adipocytes [79]. In this research, it shows us a possibility of circRNA working in two directions simultaneously. It is going to be a new perspective in our future research.

### 5.7. CiRS-133-miR-133-RDM16

Exosomes play a key role in mediating signaling transduction between neighboring or distant cells by delivering microRNAs, proteins, lncRNAs, circRNAs, and DNAs [107]. A recent study has indicated that circRNA is enriched and stable in exosomes [108]. One exosome delivered circRNA, hsa_circ_0010522 (also named ciRS-133), can suppress specific adipose miR-133 levels in preadipocytes by adsorptive action [80]. PR domain containing 16 (PRDM16), a zinc finger transcription factor controlling a brown fat/skeletal muscle switch, has been proposed to be a bidirectional cell fate switch. It promotes brown adipose tissue (BAT) differentiation while inhibits myogenesis in myoblasts [109, 110]. Loss of PRDM16 function results in myogenic differentiation of preadipocytes isolated from BAT, while the gain of PRDM16 function leads to the genesis of BAT in myoblasts [111]. PRDM16 has also been found to be a determining factor of beige adipocytes in subcutaneous white adipose tissue (WAT) and promote browning of WAT in gastric tumors [111, 112]. Previous studies have confirmed that miR-133 is the upstream regulator of PRDM16, and the miR-133/PRDM16 axis controls the formation of BAT and is linked to energy balance [7, 113]. By activating PRDM16 and suppressing miR-133, ciRS-133 activates uncoupling protein 1 (UCP1) and promotes the differentiation of preadipocytes into brown-like cells. Therefore, by targeting the miR-133/PRDM16 pathway, circulating exosomal ciRS-133 may be a common regulator that promotes white adipose browning of preadipocyte in WAT [80].

### 5.8. circRNA-0046366–miR-34a–PDGFR*α*

Studies reveal that circRNA-0046366 inhibits hepatocellular steatosis by normalizing PPAR signaling. Besides, circRNA-0046366 can antagonize the activity of miR-34a via meiotic recombination- (MRE-) based complementation [81]. miR-34a will inhibit adipogenesis by targeting PDGFR*α* [82]. By sponging miR-34a, circRNA-0046366 is associated with triglyceride metabolism at both transcriptional and translational levels. Consequently, circRNA-0046366 promotes the adipogenic differentiation through activating the ERK signaling pathway [81, 82].

### 5.9. CircSAMD4A–miR-138-5p–EZH2

Experiments indicate that circSAMD4A also named hsa_circ_0004846 regulate preadipocyte differentiation by sponging miR-138-5p. Previously, miR-138-5p has been reported targeting various proteins associated with adipogenesis in MSCs [114]. Binding to miR-138-5p, CircSAMD4A can increase the expression of EZH2 which has two downstream targets Wnt10b and Wnt1. Thus, circSAMD4A can induce adipocyte differentiation via the canonical Wnt signaling pathway [83]. It suggests that circSAMD4A can serve as a potential prognostic marker or treatment target for the therapy of tumors or metabolic diseases.

## 6. Potential circRNA Regulatory Path

As described above, circRNAs can sponge miRNA or protein to play the regulation role in adipogenesis. In general, circRNAs participate in adipogenic differentiation by modulating PPAR or Wnt pathway. Besides those targeted factors as mentioned above, there are still potential targets that may be regulated by circRNAs.

### 6.1. CircPVT1–miR-125–PPAR*α* and PPAR*γ*

CircPVT1, also known as circ6, is generated from exon 2 of the PVT1 gene. It is located on chromosome 8q2. As a homologous gene of the long noncoding RNA PVT1 (human genome GRch38/hg38), this circRNA plays a critical role in regulating human physiological and pathological functions. CircPVT1 is a senescence-associated circRNA showing markedly reduced levels in senescent fibroblasts [115]. By sponging the miR-125 family, CircPVT1 exhibits elevated levels in dividing cells and promotes cell proliferation [116]. The physiological functions of circPVT1 in gastric cancer cells include cell proliferation, cell apoptosis, and stem cell self-renewal [117]. On the launch signal of adipogenesis, c-Fos can bind to circPVT1 at its promoter region and promote the direct interaction between circPVT1 and miR-125 [116]. Studies have suggested that miR-125 can enhance the proliferation and differentiation of ADSCs [99]. The expression of miR-125 is significantly changed at day 8 after adipogenic induction. It can dramatically reduce the mRNA expression of adipogenic markers C/EBP*α*, PPAR*γ*, FABP4, fatty acid synthase (FASN), lipoprotein lipase (LPL), aP2, and estrogen-related receptor *α* (ERR*α*) [118]. Furthermore, miR-125 can inhibit the differentiation of preadipocytes by directly targeting KLF13 and affect the fatty acid composition in adipocytes by regulating elongase of very-long-chain fatty acids 6 (ELOVL6) [119]. In summary, circPVT1 may be a potential regulatory factor of adipogenesis, which simultaneously upregulates PPAR*α* and PPAR*γ* by affecting miR-125.

### 6.2. Circ-0004194

Circ-0004194 expressed in a variety of human tissues is also called Circ*β*-catenin. It can be translated into a novel 370-amino acid *β*-catenin isoform that was termed “*β*-catenin-370aa.” As the linear *β*-catenin mRNA transcript, circ*β*-catenin uses the same start codon but its translation is terminated at a new stop codon created by circularization. A recent study demonstrates that this novel isoform can stabilize full-length *β*-catenin by antagonizing GSK3*β*-induced *β*-catenin phosphorylation and degradation. Thus circ*β*-catenin potentiates the activation of the Wnt/*β*-catenin pathway in liver cancer [49]. Perhaps, circ*β*-catenin can play the same role in adipogenesis, even in cell differentiation of MSCs. Protein coding by circRNA plays a significant role in adipogenesis but rare of them has been discovered. There is still a blank area waiting for exploration, especially in the regulation of MSC differentiation.

### 6.3. CircCDK13–miR-135b-5p–PI3K/AKT and JAK/STAT

CircCDK13 (hsa_circ_0001699), a novel circRNA transcribed from the human CDK13 gene, is closely related to cell senescence and regulation of cell cycle [120]. CircCDK13 may upregulate the relevant gene expression of the signaling pathway through sponge miR-135b-5p. Therefore, circCDK13 can inhibit the PI3K/AKT pathway and JAK/STAT signaling pathway which is a very critical regulatory machinery for cellular development and proliferation in liver cancer [121].

Beyond that, by repressing miR-9, circSMAD2 impedes the activation of STAT3 and MEK/ERK pathways in migration and epithelial-mesenchymal transition [122]. CircRNA-0044073 promotes the proliferation of cells by sponging miR-107 and activating the JAK/STAT signaling pathway [123]. There are still many factors that regulate adipogenesis which may be the targets of circRNAs, such as miR-143 [124], miR-130 [125], miR-145 [126], miR-181a [127], and let-7 [128].

## 7. Conclusion

**Figure 1:**
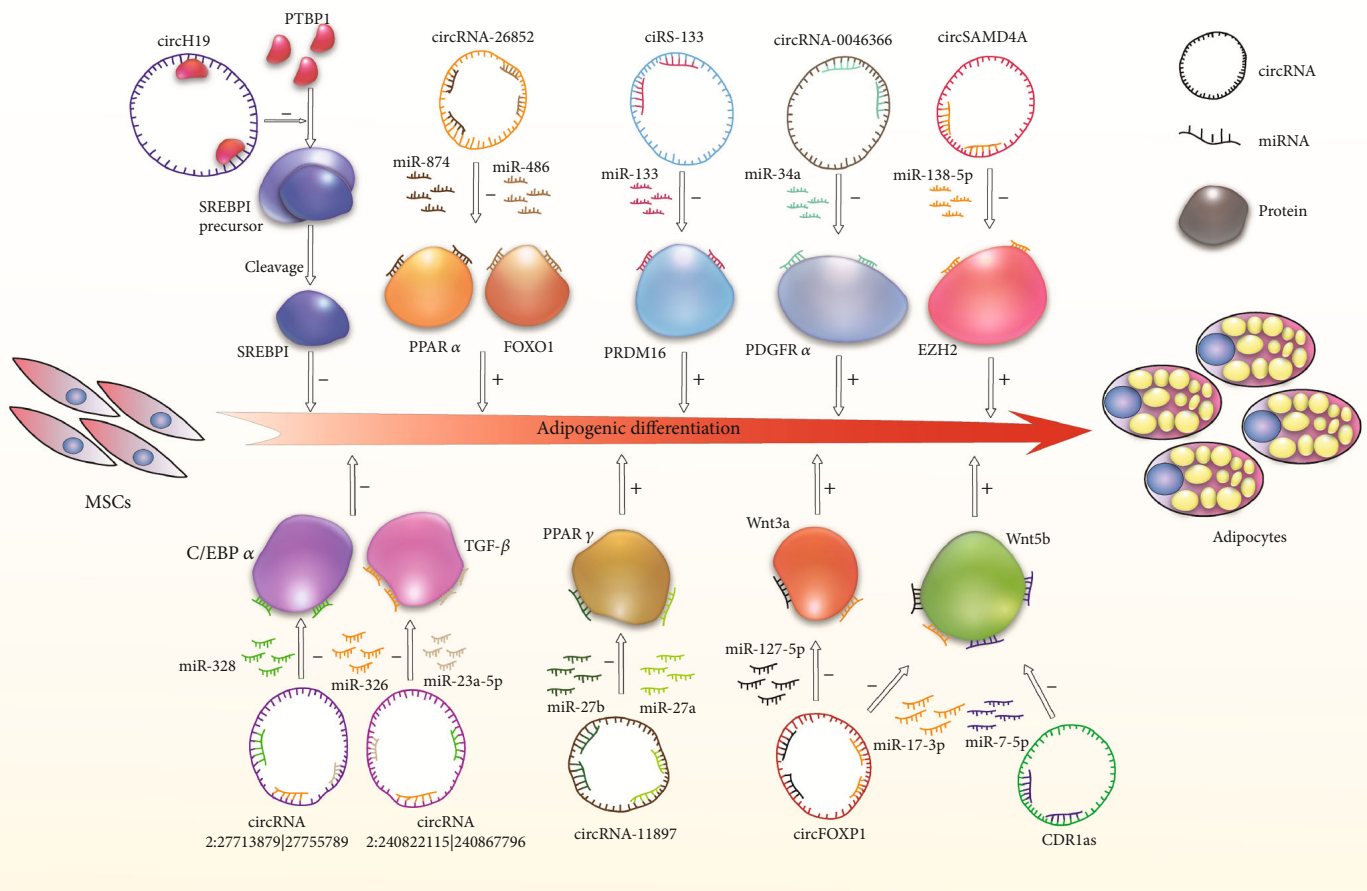
Circular RNAs regulate adipogenic differentiation.

Recent studies demonstrate that a large number of endogenous circRNAs have a major functional role in stem cell fate decision-making processes such as adipogenic differentiation. Therefore, we concluded the previous work into a primary molecular network on the regulation role of the circRNAs in adipogenesis of MSCs (Figure 1). The findings from circRNA investigations during adipogenesis suggest the potential application of circRNAs or target miRNAs to treat lipid metabolism, bone diseases, and metabolic disorders. The molecular mechanism of circFOXP1 in MSCs has been deeply discussed. It should be regarded as an essential gatekeeper of pivotal stem cell molecular networks and controlled MSC identification. With this being fundamental, circRNAs regulating cell differentiation can sum into a network which is helpful for clinical transformation. Exosomes are secreted by many different types of cells, which regulate cellular function by enabling cell-to-cell transfer of biologically active molecule, such as miRNA and circRNA. CiRS-133 in exosomes is closely linked with the browning of white adipose tissue by activating PRDM16 and suppressing miR-133. It not only provides a potential target for therapy but also lightens us with a possibility that exosomes serve as a messenger to deliver circRNAs into cells for clinical treatment.

While circRNAs are an epigenetic regulator, their potential role in modulating differentiation of MSCs is infinite. Current studies on circRNAs in MSCs' adipogenesis concentrate on the regulation of triglyceride accumulation. However, the study on circRNA regulating the commitment step of adipogenesis is still a blank field. The modes of circRNA regulating adipogenesis in existing reports are also unitary. Previous experiments mainly focused on circRNAs' function as sponge; the other functions should be explored in future study. To further elucidate the mechanisms of circRNAs on adipogenesis in stem cells, future work should be conducted to expose their binding sites to the corresponding miRNAs.

The functions of circRNAs in regulating adipogenesis have a considerable reference value in the epigenetic regulation of cell differentiation. Future investigation on circRNAs has great potential usefulness and clinical transformation.

## Figures and Tables

**Table 1:** Circular RNAs regulate adipogenic differentiation.

circRNA	miRNA or protein	Target gene(s)	Cell	Related process	Reference
Hsa_circ_0001946 (CDR1as)	miR-7-5p	Wnt5b	BMSCs	↑Adipogenesis ↓Osteogenesis	[61]
Hsa_circ_0095570 (circH19)	PTBP1	SREBP1	hADSCs	↓Adipogenesis	[53]
CircFOXP1	miR-17-3p and miR-127-5p	Wnt5b and Wnt3a	BMSCs	↑Adipogenesis	[76]
circRNA 2: 27713879|27755789 and circRNA 2: 240822115|240867796	miR-328	C/EBP*α*	MC3T3-E1	↓Adipogenesis ↑Osteogenesis	[77, 78]
	miR-23a-5p and miR-326	TGF-*β*			
circRNA-11897	miR-27a and miR-27b	PPAR*γ*	Adipocytes	↑Adipogenesis	[79]
circRNA-26852	miR-874	PPAR*α*	Adipocytes	↑Adipogenesis	[79]
	miR-486	FOXO1			
CiRS-133	miR-133	PRDM16	Preadipocytes	↑WAT browning	[80]
circRNA-0046366	miR-34a	PDGFR*α*	Adipocytes	↑Hepatocellular steatosis	[81, 82]
CircSAMD4A	miR-138-5p	EZH2	Preadipocytes	↑Adipogenesis	[83]
